# Supplementary material for: Association of Germline Variation in Driver Genes with Breast Cancer Risk in Chilean Population
Source: Int J Mol Sci. 2023 Nov 8;24(22):16076. doi: 10.3390/ijms242216076 (PMC10671568; doi:10.3390/ijms242216076)
Supplement: Supplementary file 1 [file ijms-24-16076-s001.zip › Sup Table 1.pdf]

Supplementary Table 1. Genotype and allelic frequencies of rs75704921 (*ARID2*), rs2229032 (*ATR*), rs3735156 (*KMT2C*), rs2276738 (*MAP3K13*), rs2293906 (*MAP3K13*), rs4075943 (*MAP3K13*), rs13091808 (*MAP3K13*), rs178831 (*NCOR1*), and rs3759173 (*TBX3*) in *BRCA1*/2-negative breast cancer cases and controls.

Genotype or allele	Controls (%) (n=1285)	All BC cases (n=492)			Families with ≥2 BC and/or OC cases (n=316)			Families with a single case, diagnosis at ≤50 years of age (n=176)		
		BC cases (%)	OR [95% CI]	p-value[a]	BC cases (%)	OR [95% CI]	p-value[a]	BC cases (%)	OR [95% CI]	p-value[a]
rs75704921 (*ARID2*)										
C/C	1235 (96.1)	469 (95.4)	1.0 (ref)	-	300 (94.9)	1.0 (ref)	-	170 (96.4)	1.0 (ref)	-
C/T	48 (3.7)	23 (4.6)	1.2 [0.7-2.0]	0.48	16 (5.1)	1.3 [0.7-2.5]	0.32	6 (3.6)	0.9 [0.4-2.2]	1.00
T/T	2 (0.2)	0 (0.0)	0.5 [0.02-11.4]	1.00	0 (0.0)	0.8 [0.04-18.0]	1.00	0 (0.0)	1.4 [0.07-31.3]	1.00
C/T+T/T	50 (3.9)	23 (4.6)	1.1 [0.6-1.9]	0.58	16 (5.1)	1.3 [0.7-2.3]	0.33	6 (3.6)	0.9 [0.3-2.1]	1.00
Allele C	2518 (98.0)	961 (97.7)	1.0 (ref)	-	616 (97.4)	1.0 (ref)	-	346 (98.2)	1.0 (ref)	-
Allele T	52 (2.0)	23 (2.3)	0.9 [0.7-1.0]	0.34	16 (2.6)	0.8 [0.7-1.0]	0.24	6 (1.8)	0.9 [0.7-1.2]	0.94
rs2229032 (*ATR*)										
C/C	1010 (78.6)	382 (77.6)	1.0 (ref)	-	245 (77.5)	1.0 (ref)	-	137 (77.8)	1.0 (ref)	-
C/T	261 (20.3)	101 (20.5)	1.0 [0.7-1.3]	0.89	67 (21.2)	1.0 [0.7-1.4]	0.74	34 (19.3)	0.9 [0.6-1.4]	0.91
T/T	14 (1.1)	9 (1.9)	1.8 [0.7-4.2]	0.22	4 (1.3)	1.2 [0.4-3.7]	0.75	5 (2.8)	2.7 [1.0-7.3]	0.06
C/T+T/T	275 (21.4)	110 (22.4)	1.0 [0.8-1.3]	0.64	71 (22.5)	1.0 [0.7-1.4]	0.69	39 (22.2)	1.0 [0.7-1.5]	0.84
Allele C	2281 (88.8)	865 (87.9)	1.0 (ref)	-	557 (88.1)	1.0 (ref)	-	308 (87.5)	1.0 (ref)	-
Allele T	289 (11.2)	119 (12.1)	1.0 [0.8-1.3]	0.43	75 (11.9)	1.0 [0.8-1.4]	0.61	44 (12.5)	1.1 [0.8-1.5]	0.47
rs3735156 (*KMT2C*)										
C/C	1277 (99.4)	490 (99.6)	1.0 (ref)	-	314 (99.3)	1.0 (ref)	-	176 (100)	1.0 (ref)	-
C/G	8 (0.6)	2 (0.4)	0.6 [0.1-2.7]	1	2 (0.7)	1.0 [0.2-4.3]	1	0 (0.0)	-	-
G/G	0 (0.0)	0 (0.0)	-	-	0 (0.0)	-	-	0 (0.0)	-	-
C/G+G/G	8 (0.6)	2 (0.4)	0.6 [0.14-2.7]	1	2 (0.7)	1.0 [0.2-4.3]	1	0 (0.0)	-	-
Allele C	2532 (99.7)	982 (99.8)	1.0 (ref)	-	630 (99.7)	1.0 (ref)	-	352 (100)	1.0 (ref)	-
Allele G	8 (0.3)	2 (0.2)	0.6 [0.1-2.7]	1	2 (0.3)	1.0 [0.2-4.3]	1	0 (0.0)	-	-
rs2276738 (*MAP3K13*)										
G/G	432 (33.6)	160 (32.5)	1.0 (ref)	-	92 (29.1)	1.0 (ref)	-	67 (38.3)	1.0 (ref)	-
G/C	612 (47.6)	225 (45.7)	0.9 [0.7-1.2]	0.98	157 (49.7)	1.2 [0.8-1.5]	0.26	69 (38.9)	0.7 [0.5-1.0]	0.09
C/C	241 (18.8)	107 (21.8)	1.1 [0.8-1.6]	0.23	67 (21.3)	1.3 [0.9-1.8]	0.15	40 (22.8)	1.0 [0.6-1.6]	0.82
G/C+C/C	1044 (66.4)	332 (67.5)	0.8 [0.6-1.0]	0.17	224 (70.9)	1.2 [0.9-1.6]	0.16	109 (61.7)	0.8 [0.5-1.1]	0.22
Allele G	1476 (57.4)	545 (55.4)	1.0 (ref)	-	341 (54.0)	1.0 (ref)	-	203 (57.9)	1.0 (ref)	-
Allele C	1094 (42.6)	439 (44.6)	1.0 [0.9-1.2]	1.0	291 (46.0)	1.1 [0.9-1.3]	0.14	149 (42.1)	0.9 [0.7-1.2]	0.90
rs2293906 (*MAP3K13*)										

C/C	420 (32.7)	171 (34.7)	1.0 (ref)	-	106 (33.6)	1.0 (ref)	-	65 (36.8)	1.0 (ref)	-
C/T	617 (48.0)	234 (47.6)	0.9 [0.7-1.1]	0.584	163 (51.6)	1.0 [0.7-1.3]	0.776	71 (40.4)	0.7 [0.5-1.0]	0.131
T/T	248 (19.3)	87 (17.7)	0.8 [0.6-1.1]	0.351	47 (14.8)	0.7 [0.5-1.0]	0.152	40 (22.8)	1.0 [0.6-1.6]	0.826
C/T+T/T	865 (67.3)	321 (65.3)	0.9 [0.7-1.1]	0.422	210 (66.4)	0.9 [0.7-1.2]	0.785	111 (63.2)	0.8 [0.5-1.1]	0.298
Allele C	1457 (56.7)	576 (58.5)	1.0 (ref)	-	375 (59.4)	1.0 (ref)	-	201 (57.0)	1.0 (ref)	-
Allele T	1113 (35.7)	408 (41.5)	0.9 [0.7-1.0]	0.345	257 (40.6)	0.8 [0.7-1.0]	0.245	151 (43.0)	0.9 [0.7-1.2]	0.949
rs4075943 (*MAP3K13*)										
T/T	841 (65.4)	323 (65.7)	1.0 (ref)	-	212 (67.2)	1.0 (ref)	-	111 (63.0)	1.0 (ref)	-
T/A	402 (31.3)	148 (30.0)	0.9 [0.7-1.2]	0.72	94 (29.6)	0.9 [0.7-1.2]	0.58	54 (30.6)	1.0 [0.7-1.4]	0.92
A/A	42 (3.3)	21 (4.3)	1.3 [0.7-2.2]	0.31	10 (3.2)	0.9 [0.4-1.9]	1.00	11 (6.4)	1.9 [0.9-3.9]	0.07
T/A+A/A	444 (34.6)	169 (34.3)	0.9 [0.7-1.2]	0.95	104 (32.8)	0.9 [0.7-1.2]	0.59	65 (37.0)	1.1 [0.7-1.5]	0.55
Allele T	2084 (81.1)	794 (80.7)	1.0 (ref)	-	518 (82.0)	1.0 (ref)	-	276 (78.3)	1.0 (ref)	-
Allele A	486 (18.9)	190 (19.3)	1.0 [0.8-1.2]	0.82	114 (18.0)	0.9 [0.7-1.1]	0.63	76 (21.7)	1.1 [0.9-1.5]	0.25
rs13091808 (*MAP3K13*)										
C/C	956 (74.4)	370 (75.2)	1.0 (ref)	-	237 (75.0)	1.0 (ref)	-	133 (75.6)	1.0 (ref)	-
C/T	297 (23.1)	111 (22.6)	0.9 [0.7-1.2]	0.84	72 (22.9)	0.9 [0.7-1.3]	1.00	39 (22.1)	0.9 [0.6-1.4]	0.84
T/T	32 (2.5)	11 (2.2)	0.8 [0.4-1.8]	0.85	7 (2.1)	0.8 [0.3-2.0]	1.00	4 (2.3)	0.9 [0.3-2.8]	1.00
C/T+T/T	329 (25.6)	122 (24.8)	0.9 [0.7-1.2]	0.79	79 (25.0)	0.9 [0.7-1.3]	0.88	43 (24.4)	0.9 [0.6-1.3]	0.84
Allele C	2209 (86.0)	851 (86.5)	1.0 (ref)	-	546 (86.5)	1.0 (ref)	-	305 (86.5)	1.0 (ref)	-
Allele T	361 (14.0)	133 (13.5)	0.9 [0.7-1.1]	0.75	86 (13.5)	0.9 [0.7-1.2]	0.81	47 (13.5)	0.9 [0.6-1.3]	0.86
rs178831 (*NCOR1*)										
G/G	452 (35.2)	197 (40.1)	1.0 (ref)	-	129 (41.0)	1.0 (ref)	-	68 (38.7)	1.0 (ref)	-
G/A	624 (48.5)	225 (45.7)	0.8 (0.6-1.0)	0.10	141 (44.6)	0.7 (0.6-1.0)	0.10	84 (47.6)	0.8 (0.6-1.2)	0.53
A/A	209 (16.3)	70 (14.2)	0.7 (0.5-1.0)	0.12	46 (14.4)	0.7 (0.5-1.1)	0.18	24 (13.7)	0.7 (0.4-1.2)	0.32
G/A+A/A	833 (64.8)	295 (59.9)	0.8 (0.6-1.0)	0.06	187 (59.1)	0.7 (0.6-1.0)	0.07	108 (61.3)	0.8 (0.6-1.1)	0.39
Allele G	1528 (59.5)	619 (63)	1.0 (ref)	-	399 (63.3)	1.0 (ref)	-	220 (62.5)	1.0 (ref)	-
Allele A	1042 (40.5)	365 (37)	0.8 (0.7-1.0)	0.06	233 (36.7)	0.8 (0.7-1.0)	0.09	132 (37.5)	0.8 (0.6-1.1)	0.31
rs3759173 (*TBX3*)										
C/C	1077 (83.8)	409 (83.1)	1.0 (ref)	-	261 (82.5)	1.0 (ref)	-	148 (84.1)	1.0 (ref)	-
C/A	199 (15.5)	81 (16.5)	1.0 [0.8-1.4]	0.61	53 (16.9)	1.1 [0.7-1.5]	0.54	28 (15.9)	1.0 [0.6-1.5]	0.91
A/A	9 (0.7)	2 (0.4)	0.5 [0.1-2.7]	0.73	2 (0.6)	0.9 [0.1-4.2]	1.00	0 (0.0)	0.3 [0.02-6.5]	0.61
C/A+A/A	208 (16.2)	83 (16.9)	1.0 [0.7-1.3]	0.72	55 (17.5)	1.0 [0.7-1.5]	0.61	28 (15.9)	0.9 [0.6-1.5]	1.00
Allele C	2353 (91.6)	899 (91.4)	1.0 (ref)	-	575 (91.0)	1.0 (ref)	-	324 (92.0)	1.0 (ref)	-
Allele A	217 (8.4)	85 (8.6)	1.0 [0.7-1.3]	0.88	57 (8.0)	1.0 [0.7-1.4]	0.67	28 (8.0)	0.9 [0.6-1.4]	0.82

BC breast cancer, *OC* ovarian cancer, *OR* odds ratio, *CI* confidence interval; [a] Fisher's exact test; Bold values are statistically significant (p<0.05).
