# Supplementary material for: Association of Germline Variation in Driver Genes with Breast Cancer Risk in Chilean Population
Source: Int J Mol Sci. 2023 Nov 8;24(22):16076. doi: 10.3390/ijms242216076 (PMC10671568; doi:10.3390/ijms242216076)
Supplement: Supplementary file 1 [file ijms-24-16076-s001.zip › Sup Table 2.pdf]

Supplementary Table 2. Genotype and allelic frequencies of rs75704921 (*ARID2*), rs2229032 (*ATR*), rs3735156 (*KMT2C*), rs2276738 (*MAP3K13*), rs2293906 (*MAP3K13*), rs4075943 (*MAP3K13*), rs13091808 (*MAP3K13*), rs178831 (*NCOR1*), and rs3759173 (*TBX3*) according the number of BC cases in the families in *BRCA1*/2-negative breast cancer cases and controls.

Genotype or allele	Controls (%) (n=1258)	Families with 2 BC and/or OC cases (n=165)			Families with ≥ 3 BC and/or OC cases (n=151)		
		BC cases (%)	OR [95% CI]	*P* value[a]	BC cases (%)	OR [95% CI]	*P* value[a]
rs75704921 (*ARID2*)							
C/C	1235 (96.1)	156 (94.8)	1.0 (ref)	-	143 (94.9)	1.0 (ref)	-
C/T	48 (3.7)	9 (5.2)	1.0 [0.7-1.4]	0.92	8 (5.1)	1.0 [0.7-1.5]	0.76
T/T	2 (0.2)	0 (0.0)	0.7 [0.4-1.1]	0.21	0 (0.0)	0.7 [0.4-1.3]	0.43
C/T+T/T	50 (3.9)	9 (5.2)	0.9 [0.6-1.3]	0.72	8 (5.1)	0.9 [0.6-1.4]	1.00
Allele C	2518 (98.0)	21 (97.4)	1.0 (ref)	-	294 (97.5)	1.0 (ref)	-
Allele T	52 (2.0)	9 (2.6)	0.8 [0.6-1.1]	0.29	8 (2.5)	0.9 [0.7-1.1]	0.36
rs2229032 (*ATR*)							
C/C	1010 (78.6)	124 (74.8)	1.0 (ref)	-	122 (80.4)	1.0 (ref)	-
C/T	261 (20.3)	39 (23.9)	1.2 [0.8-1.8]	0.29	27 (18.2)	0.8 [0.5-1.3]	0.65
T/T	14 (1.1)	2 (1.3)	1.2 [0.2-4.8]	0.67	2 (1.4)	1.3 [0.2-5.0]	0.66
C/T+T/T	275 (21.4)	41 (25.2)	1.2 [0.8-1.8]	0.30	28 (19.6)	0.8 [0.5-1.3]	0.66
Allele C	2281 (88.8)	287 (86.8)	1.0 (ref)	-	271 (89.5)	1.0 (ref)	-
Allele T	289 (11.2)	43 (13.2)	1.2 [0.8-1.7]	0.30	31 (10.5)	0.9 [0.6-1.3]	0.76
rs3735156 (*KMT2C*)							
C/C	1277 (99.4)	164 (99.4)	1.0 (ref)	-	150 (99.3)	1.0 (ref)	-
C/G	8 (0.6)	1 (0.6)	1.01 [0.09-6.59]	1	1 (0.7)	1.13 [0.10-7.37]	0.610
G/G	0 (0.0)	0 (0)	-	-	0 (0)	-	-
C/G+G/G	8 (0.6)	1 (0.6)	1.01 [0.09-6.59]	1	1 (0.7)	1.13 [0.10-7.37]	0.610
Allele C	2532 (99.7)	329 (99.7)	1.0 (ref)	-	301 (99.6)	1.0 (ref)	-
Allele G	8 (0.3)	1 (0.3)	1.01 [0.09-6.53]	1	1 (0.4)	1.13 [0.10-7.29]	0.609
rs2276738 (*MAP3K13*)							
G/G	432 (33.6)	52 (31.4)	1.0 (ref)	-	40 (26.4)	1.0 (ref)	-
G/C	612 (47.6)	75 (45.5)	1.0 [0.6-1.4]	1.00	82 (54.3)	1.4 [0.9-2.1]	0.12
C/C	241 (18.8)	38 (23.1)	1.3 [0.8-2.0]	0.24	29 (19.3)	1.2 [0.7-2.1]	0.34
G/C+C/C	1044 (66.4)	113 (68.6)	0.8 [0.6-1.2]	0.58	111 (73.6)	1.3 [0.9-2.0]	0.10
Allele G	1476 (57.4)	179 (54.2)	1.0 (ref)	-	162 (53.6)	1.0 (ref)	-
Allele C	1094 (42.6)	151 (45.8)	1.1 [0.8-1.4]	0.32	140 (46.4)	1.1 [0.9-1.4]	0.26
rs2293906 (*MAP3K13*)							

C/C	420 (32.7)	56 (34.2)	1.0 (ref)	-	50 (32.9)	1.0 (ref)	-
C/T	617 (48.0)	85 (51.6)	1.0 [0.7-1.4]	0.92	78 (51.7)	1.0 [0.7-1.5]	0.76
T/T	248 (19.3)	24 (14.3)	0.7 [0.4-1.1]	0.21	23 (15.4)	0.7 [0.4-1.3]	0.43
C/T+T/T	865 (67.3)	109 (65.8)	0.9 [0.6-1.3]	0.72	101 (67.1)	0.9 [0.6-1.4]	1.00
Allele C	1457 (56.7)	197 (59.9)	1.0 (ref)	-	178 (58.7)	1.0 (ref)	-
Allele T	1113 (35.7)	133 (40.1)	0.8 [0.6-1.1]	0.29	124 (41.3)	0.9 [0.7-1.1]	0.36
rs4075943 (*MAP3K13*)							
T/T	841 (65.4)	103 (62.6)	1.0 (ref)	-	109 (72.3)	1.0 (ref)	-
T/A	402 (31.3)	56 (33.7)	1.1 [0.7-1.6]	0.52	38 (25.0)	0.7 [0.4-1.0]	0.10
A/A	42 (3.3)	6 (3.7)	1.1 [0.4-2.8]	0.63	4 (2.7)	0.7 [0.2-2.1]	0.81
T/A+A/A	444 (34.6)	62 (37.4)	1.1 [0.8-1.5]	0.48	42 (27.7)	0.7 [0.5-1.0]	0.10
Allele T	2084 (81.1)	262 (79.4)	1.0 (ref)	-	256 (84.8)	1.0 (ref)	-
Allele A	486 (18.9)	68 (20.6)	1.1 [0.8-1.4]	0.53	46 (15.2)	0.7 [0.5-1.0]	0.13
rs13091808 (*MAP3K13*)							
C/C	956 (74.4)	125 (75.8)	1.0 (ref)	-	112 (74.1)	1.0 (ref)	-
C/T	297 (23.1)	37 (22.2)	0.9 [0.6-1.4]	0.83	36 (23.7)	1.0 [0.6-1.5]	0.91
T/T	32 (2.5)	3 (2.0)	0.7 [0.2-2.6]	1.00	3 (2.2)	0.8 [0.2-2.9]	1.00
C/T+T/T	329 (25.6)	40 (24.2)	0.9 [0.6-1.3]	0.76	39 (25.9)	1.0 [0.6-1.5]	0.91
Allele C	2209 (86.0)	287 (86.9)	1.0 (ref)	-	260 (86.0)	1.0 (ref)	-
Allele T	361 (14.0)	43 (13.1)	0.9 [0.6-1.3]	0.71	42 (14.0)	1.0 [0.7-1.4]	0.93
rs178831 (*NCOR1*)							
G/G	452 (35.2)	69 (41.8)	1.0 (ref)	-	60 (40)	1.0 (ref)	-
G/A	624 (48.5)	74 (44.9)	0.7 (0.5-1.1)	0.19	67 (44.3)	0.8 (0.5-1.1)	0.28
A/A	209 (16.3)	22 (13.3)	0.6 (0.4-1.1)	0.17	24 (15.7)	0.8 (0.5-1.4)	0.60
G/A+A/A	833 (64.8)	96 (58.2)	0.7 (0.5-1.0)	0.11	91 (60.0)	0.8 (0.5-1.1)	0.26
Allele G	1528 (59.5)	212 (64.2)	1.0 (ref)	-	187 (62.1)	1.0 (ref)	-
Allele A	1042 (40.5)	118 (35.8)	0.8 (0.6-1.0)	0.11	115 (37.9)	0.8 (0.6-1.1)	0.40
rs3759173 (*TBX3*)							
C/C	1077 (83.8)	139 (84.3)	1.0 (ref)	-	121 (80.4)	1.0 (ref)	-
C/A	199 (15.5)	24 (14.5)	0.9 [0.5-1.4]	0.81	30 (19.6)	1.3 [0.8-2.0]	0.23
A/A	9 (0.7)	2 (1.2)	1.7 [0.3-7.9]	0.37	0 (0.0)	0.4 [0.02-8.1]	1.00
C/A+A/A	208 (16.2)	26 (15.7)	0.9 [0.6-1.4]	0.91	30 (19.6)	1.2 [0.8-1.9]	0.29
Allele C	2353 (91.6)	302 (91.5)	1.0 (ref)	-	272 (90.1)	1.0 (ref)	-
Allele A	217 (8.4)	28 (8.5)	0.9 [0.6-1.5]	0.92	30 (9.9)	1.1 [0.7-1.7]	0.50

BC breast cancer, *OC* ovarian cancer, *OR* odds ratio, *CI* confidence interval, ref: Reference; [a] Fisher's exact test.
